# The Right Moment to Unload: Preventive Single Inflow Cannula LAVA-ECMO and the Timing of Left Ventricular Decompression in Cardiogenic Shock

**DOI:** 10.1016/j.jscai.2026.105680

**Published:** 2026-09-10

**Authors:** Ahmad Jabri, Gennaro Giustino, Pedro A. Villablanca

**Affiliations:** a Division of Cardiovascular Medicine, William Beaumont University Hospital, Royal Oak, Michigan; b Gagnon Cardiovascular Institute, Atlantic Health System, Morristown, New Jersey; c Division of Cardiovascular Medicine, Henry Ford Hospital, Detroit, Michigan

**Keywords:** Cardiogenic shock, LAVA-ECMO, mechanical circulatory support

Left ventricular (LV) overload is the recurring liability of peripheral veno-arterial extracorporeal membrane oxygenation (VA-ECMO). The retrograde aortic flow that restores systemic perfusion simultaneously raises LV afterload, promoting distension, myocardial wall stress, pulmonary congestion, and stasis-related thrombosis.^1^ For more than a decade, the field has agreed on the what—that unloading the pressurized ventricle is desirable^2^^,^^3^—while continuing to argue about the whom, the when, and the how. In this issue of *JSCAI*, Shih et al^4^ offer a provocative perspective on the timing of ventricular unloading and a technically elegant answer to the how.

The existing evidence, however, remains conflicted. The 2 randomized trials that directly tested early left atrial (LA) decompression—EARLY-UNLOAD^5^ and EVOLVE-ECMO^6^—confirmed that transseptal LA drainage relieves congestion and improves aortic valve opening, yet neither demonstrated a survival benefit over a conventional watch-and-rescue approach. Against that neutral signal sits DanGer Shock, in which a microaxial flow pump reduced mortality in infarct-related shock in select patients,^7^ reminding us that the intensity and timing of ventricular unloading may matter as much as the decision to unload at all. The unresolved tension between these results is precisely the space this single-center cohort tries to occupy.

Shih et al^4^ studied a “preventive” strategy: an a priori commitment to LA unloading at the moment of ECMO initiation, delivered through a single inflow cannula advanced transeptally so that its side holes drain both atria while the tip sits in the left atrium or left superior pulmonary vein—either primarily or by planned conversion from VA-ECMO within 60 minutes. Forty-two patients managed this way were compared with 64 contemporaneous patients supported by conventional VA-ECMO. The conceptual innovation of this study lies less in the cannula configuration itself than in the treatment strategy it represents. Rather than reserving left-sided decompression for patients who develop ventricular distension or pulmonary edema after VA-ECMO initiation, the investigators adopted a protocol of preventive unloading at the time of cannulation. This shift—from reactive to preventive decompression—is what distinguishes the present study from previous investigations and provides the rationale for testing whether timing itself influences clinical outcomes.

The reported results are provocative and hypothesis generating.^4^ Thirty-day all-cause mortality was 47.6% with preventive LAVA-ECMO vs 76.6% with VA-ECMO (adjusted hazard ratio, 0.54; 95% CI, 0.31-0.95). Signs of LV overload were roughly a third as frequent (adjusted odds ratio, 0.19; 95% CI, 0.08-0.49), failure to wean trended lower, and overall complication rates were comparable, with the notable exception of 2 right atrial ruptures in the LAVA arm. A 4-group analysis suggested a stepwise mortality gradient tracking the intensity of unloading, with the lowest event rates in patients receiving both LA drainage and an intra-aortic balloon pump.

These findings deserve enthusiasm tempered by discipline, and to the authors’ credit, they supply much of the discipline themselves. A nearly 30 percentage point absolute mortality difference exceeds anything a dedicated device trial has produced, and an effect of that magnitude in a nonrandomized comparison should prompt us to look first for selection rather than biology. The allocation here was operator driven, contingent on the availability of physicians comfortable with transseptal puncture—a form of confounding that no multivariable model fully neutralizes. The authors are candid that the control group is unusual: only 2 of 64 VA-ECMO patients ever received rescue mechanical unloading, whereas half to three-quarters of the control patients in EARLY-UNLOAD^5^ and EVOLVE-ECMO^6^ eventually did. Read charitably, this contrast supports the authors’ thesis that never unloading is worse than unloading early. Read skeptically, it means the comparator may represent a lower standard of contemporary care than most readers would recognize as their own, inflating the apparent benefit. The 6 percentage point excess of precannulation resuscitation in the VA arm points in the same direction.

Just as valuable is the mechanistic honesty of the discussion. In the subset with invasive measurements, LAVA-ECMO stabilized LV end-diastolic pressure at roughly 18 to 21 mm Hg—better than the rising pressures seen with VA-ECMO alone but short of the sub-15 mm Hg threshold that defines true decompression and far from the sub-5 mm Hg that a transvalvular pump can achieve. The authors, therefore, reframe their intervention accurately: this is prevention of overload rather than definitive unloading. That distinction is clinically consequential. A pulse pressure that collapses below 10 mm Hg after cannulation signals a barely ejecting ventricle and mandates vigilance for LV and aortic-root stasis—one reason the observed synergy with intra-aortic balloon pump, which restores intermittent aortic valve opening and augments coronary perfusion, is biologically coherent rather than merely statistical.

It is also important to place these findings within the broader evolution of LAVA-ECMO. Previous studies established the feasibility of LA drainage,^8^ demonstrated consistent hemodynamic improvement,^9^ and defined its role primarily in structural and valvular cardiogenic shock.^10^ Shih et al^4^ extend this experience by asking a different question—not whether LAVA-ECMO works, but whether initiating it before ventricular overload develops changes clinical outcomes. In a comprehensive review, Jabri et al^11^ frame the technique as a strategy for structural and valvular cardiogenic shock—severe aortic regurgitation, ventricular septal defect, and biventricular failure—where conventional support struggles to reconcile ventricular unloading with systemic perfusion and where LA drainage serves principally as a bridge to definitive transcatheter or surgical valve therapy. That literature documents impressive acute hemodynamics. In the largest single-center experience (68 patients), LAVA-ECMO reduced right atrial (−5 mm Hg), mean pulmonary artery (−9 mm Hg), pulmonary capillary wedge (−10 mm Hg), and LV end-diastolic (−14 mm Hg) pressures within 24 hours of cannulation, with survival to decannulation of 69% but 30-day survival and in-hospital survival of only about 52%.^9^ A dedicated valvular shock series reported comparable LV end-diastolic pressure reductions (on the order of 18-21 mm Hg) with no complications attributable to transseptal cannulation.^10^ Yet across these reports, in-hospital mortality remained high, bleeding was a recurrent hazard, and long-term outcomes and optimal patient selection stayed unresolved. Viewed against that backdrop, Shih et al^4^ represent a conceptual extension rather than a replication: they move LAVA-ECMO from a rescue-and-bridge tool in structural disease toward a preventive, protocol-level strategy in a broad, predominantly infarct-related shock population, and ask whether committing to unload up front—before overload declares itself—changes survival.

Where, then, does preventive single inflow LAVA-ECMO belong? It is unlikely to displace a microaxial flow pump where potent, direct decompression is the priority and anatomy permits it. Its niche is elsewhere—patients requiring durable, high-flow biventricular support; those with valvular anatomy hostile to a transvalvular device; and the many health systems in which a percutaneous, single-circuit, 2-access-site solution is more available and affordable than an escalating stack of hardware. In those settings, the technique is pragmatic, and the signal that earlier is better is worth taking seriously.

Whether preventive unloading should become the default strategy for all patients supported with VA-ECMO remains uncertain. The answer will likely differ according to shock phenotype, ventricular reserve, and the anticipated duration of mechanical support. Identifying the patients most likely to benefit from preventive decompression may ultimately prove as important as the unloading strategy itself.

This study offers a plausible hypothesis and a strong case for the next step: a randomized trial. That trial should compare LAVA-ECMO against a control arm that reflects real current practice—including protocol-driven rescue unloading—and should standardize transseptal expertise across sites. Until then, Shih et al^4^ have done the field a service by reminding us that in cardiogenic shock, the timing may be as important as the cannula. Whether preventive unloading ultimately changes outcomes remains to be proven, but this study appropriately shifts the conversation from how to unload to when unloading should begin.

## CRediT authorship contribution statement

**Ahmad Jabri:** Writing – original draft. **Gennaro Giustino:** Writing – review & editing. **Pedro A. Villablanca:** Writing – review & editing.

## Declaration of competing interest

Pedro A. Villablanca is a consultant for Edwards Lifesciences, Medtronic, Abiomed, Shockwave Medical, and AngioDynamics. Gennaro Giustino is a consultant for Edwards Lifesciences. Ahmad Jabri is a consultant for Edwards Lifesciences.
